# White-Nose Syndrome Fungus (*Geomyces destructans*) in Bat, France

**DOI:** 10.3201/eid1602.091391

**Published:** 2010-02

**Authors:** Sébastien J. Puechmaille, Pascal Verdeyroux, Hubert Fuller, Meriadeg Ar Gouilh, Michaël Bekaert, Emma C. Teeling

**Affiliations:** University College Dublin, Dublin, Ireland (S.J. Puechmaille, H. Fuller, M. Bekaert, E.C. Teeling); Groupe Chiroptères Aquitaine, Erdoia, France (P. Verdeyroux); Institut Pasteur, Paris, France (M. Ar Gouilh)

**Keywords:** Chiroptera, Geomyces destructans, Myotis myotis, bats, white-nose syndrome, fungi, France, hibernation, dispatch

## Abstract

White-nose syndrome is caused by the fungus *Geomyces destructans* and is responsible for the deaths of >1,000,000 bats since 2006. This disease and fungus had been restricted to the northeastern United States. We detected this fungus in a bat in France and assessed the implications of this finding.

Biologists are struggling to understand a recent emerging infectious disease, white-nose syndrome (WNS) (*1*), which potentially threatens >20% of all mammalian diversity (bats) (*2*). WNS is a deadly epidemic that has swept through the northeastern United States over the past 3 years and caused the death of >1,000,000 bats, with decreases of ≈100% in some populations (*3*).

This disease is associated with hibernating, cave-roosting bats. A visually conspicuous white fungus grows on the face, ears, or wings of stricken bats; infiltration of the hyphae into membranes and tissues leads to severe damage (*4*). Bats that exhibit WNS have little or no fat reserves, which are essential for their survival throughout and after hibernation (*5*). The fungus associated with WNS is a newly described, psychrophilic (cold-loving) species (*Geomyces destructans*) (*6*). It is closely related to *G*. *pannorum*, which causes skin infections in humans (*7*).

Although it is not known whether the fungus is primarily responsible for deaths of bats or is a secondary infection, it is directly associated with deaths of bats (*5*). Bacteriologic, virologic, parasitologic, and postmortem evaluations for the cause of death did not identify any other agents, which reinforces the suspicion that this fungus is the causative agent (*4**,**5*). To date, WNS has been found only in the northeastern United States. However, researchers have suggested its presence in Europe. We investigated whether *G*. *destructans* is present in bats in Europe and assessed the implications of its presence.

## The Study

**Figure 1:**
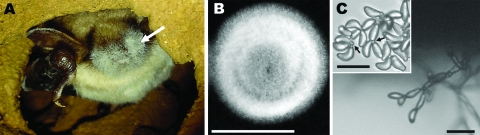
A) *Myotis myotis* bat found in a cave on March 12, 2009, in France, showing white fungal growth on its nose (arrow). B) Fungus colony on malt extract medium after incubation for 3 weeks at 10°C. Scale bar = 1 cm. C) Clusters of unstained spores of *Geomyces destructans*. Spores in the inset were stained with lactophenol cotton blue, which shows the truncate spore base (arrows) and surface granulation. Scale bars = 10 µm.

During intensive monitoring of bat hibernation in France, 1 bat (*Myotis myotis*) found on March 12, 2009, near Périgueux (45°8′N, 0°44′E), showed a powdery, white fungal growth on its nose (Figure 1, panel A), which is characteristic of WNS. Sterile dry cotton swabs were used to collect fungus material from the nose of the bat. The bat was then weighed, measured, and released. Swabs were moistened with 50 μL of sterile water and streaked onto plates containing potato dextrose agar supplemented with 0.1% mycologic peptone. Plates (9 cm in diameter) were sealed with parafilm and incubated inverted at 10°C. A dense fungus growth developed within 14 days (Figure 1, panel B).

Cultures were established by transferring inoculum to other mycologic media, including malt extract agar and Sabouraud agar. Colonies on malt extract agar were initially white but after spore production and aging they quickly darkened from the center to a dull gray, often showing a faint green hue. Spores were hyaline, irregularly curved, broadly crescent-shaped (typically 6–8 μm long and 3–4 μm wide), and narrowed at each end, one of which was broadly truncate, often showing an annular frill (Figure 1, panel C). Fungal cultures have been deposited in the culture collection of the Industrial Microbiology Department of University College Dublin (Reference IMD Z2053).

Microscopic examination of the original swab samples showed numerous spores with the above-mentioned features. The psychrophilic nature of the fungus and its species-specific morphologic features (Technical Appendix) led to the conclusion that this fungus was *G*. *destructans*, which was recently isolated from WNS-positive bats in the northeastern United States (*6*).

Two molecular markers were sequenced from 6 randomly chosen fungus cultures to confirm species identity. DNA was extracted by using a Blood and Tissue Kit (QIAGEN, Hilden, Germany) following the manufacturer’s instructions with slight modifications (after step 3, we added an incubation time of 10 min at 70°C). The internal transcribed spacer (ITS) regions (ITS1, 5.8S, and ITS2) and the small subunit (SSU) of the rRNA gene were amplified separately.

PCRs were performed in 25-μL volumes containing 1 μL of DNA (10–75 ng/μL), 1.5 mmol/L MgCl_2_, 0.4 μmol/L of each primer, 0.2 mmol/L dNTP, 1× PCR buffer, and 1 U of Platinum Taq DNA Polymerase High Fidelity (Invitrogen, Carslbad, CA, USA). Identical PCR cycling conditions were used for both fragments: an initial step at 95°C for 15 s; 10 cycles at 95°C for 30 s, 60°C (reduction of 2°C every 2 cycles) for 1 min and 45 s, and 72°C for 1 min; 30 cycles at 95°C for 30 s, 50°C for 1 min and 45 s, and 72°C for 1 min; and a final step at 72°C for 10 min. PCR products were purified and sequenced in both directions by using primers listed in the Table. Complementary sequences were assembled and edited for accuracy by using CodonCode Aligner version 3.0.3 (www.codoncode.com/aligner/download.htm).

**Table:** Primers used for PCR amplification and sequencing of fungus in bats, France*

Gene	Primer sequence (5′ → 3′)	PCR
ITS	TCCTCCGCTTATTGATATGC	Forward
	GGAAGTAAAAGTCGTAACAAGG	Reverse
SSU rRNA	CTGGTTGATTCTGCCAGT	Forward
	AAACCTTGTTACGACTTTTA	Reverse
	CCGGAGAAGGAGCCTGAGAAAC	†
	AACTTAAAGGAATTGACGGAAG	†
	CTCATTCCAATTACAAGACC	†
	GAGTTTCCCCGTGTTGAGTC	†

*ITS, internal transcribed spacer; SSU, small subunit. †Used for sequencing only.

**Figure 2:**
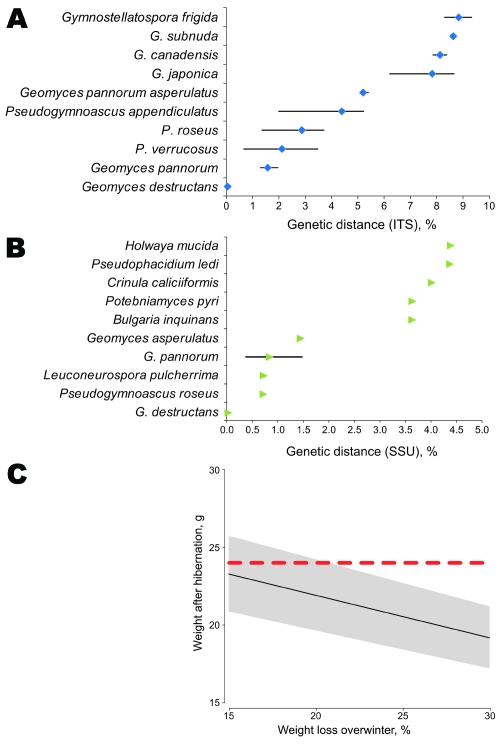
Genetic distance between fungus A) internal transcribed spacer (ITS) (474 nt) and B) small subunit (SSU) rRNA (1,865 nt) gene sequences and other closely related fungus species present in GenBank. Results are based on pairwise sequence comparisons with gaps and missing data removed. Error bars in panels A and B indicate mean ± SD. C) Estimation of weight of *Myotis myotis* bats after hibernation as a function of the range of percentage of weight loss reported. Posthibernation of a bat’s weight was estimated from prehibernation measured weights (n = 155 bats) minus winter fat loss. A strong positive relationship exists between body mass and fat mass during prehibernation (*8*). Fat reserves between 15% and 30% of body mass at the onset of hibernation have been reported to be necessary for *Myotis* species to survive winter (*9*). The posthibernation weight (W_post_) was thus estimated as W_post_ = W_pre_ – (W_pre_ × W_loss_/100), where W_pre_ is prehibernation weight and W_loss_ is percentage of body mass lost during hibernation. Mean ± SD prehibernation weight of 155 bats captured in France during August–October 2009 (27.42 ± 2.87 g) was used for the estimate. Black line represents the mean, gray area represents the mean ± SD, and red dashed line represents the 24-g weight of the bat caught in France with white-nose syndrome posthibernation. The bat was in good condition (24 g) because it weighed more than the expected average for a posthibernating bat despite having *Geomyces destructans* growth on its snout.

The ITS and SSU sequences from the 6 WNS fungus cultures were identical. They were deposited in GenBank as single sequences: ITS (GQ489024) and SSU (GQ489025). Sequences obtained for the 2 genetic markers showed a 100% sequence identity with the described *G*. *destructans* fungus (Figure 2, panels A, B). Thus, morphologic and genetic data support the presence of *G*. *destructans* infection in a bat in France.

## Conclusions

Our results show that the WNS fungus was present in a bat in France and has implications for WNS research, bat conservation, and emerging infectious disease control. We suggest 3 possible scenarios for our findings. The first scenario is that the fungus has only recently arrived in Europe and all bats in Europe are now at risk for infection. Thus, conservation steps must be taken to minimize the spread of this disease, especially because this disease is specific for hibernating bats. After the hibernation period, *M*. *myotis* bats may migrate up to 436 km to reach their summer roosts (*10*), a behavior that could quickly increase the chance of fungus transmission. A second scenario is that the fungus has been present in Europe for a long time. Because mass deaths have not been observed in bats in Europe, these bats may be immune to WNS. Therefore, identification of mechanisms of this immunity will advance understanding of this disease and fungus resistance in mammals. The third scenario is that the *G*. *destructans* fungus is not the primary cause of death but acts as an opportunistic pathogen in bats already immunocompromised by other pathogens such as viruses or bacteria (*1*). Comparison of pathogens in bats in Europe and the United States infected with *G*. *destructans* should identify the primary causative agent.

The bat in our study showing fungal growth was not underweight (Figure 2, panel C), as is typical of bats in the United States with WNS (*4*). This finding favors the second or third scenarios. Also, a 6-year (2004–2009) annual monitoring program of wintering bat populations at the site and 5 sites within a 2-km radius did not show any cases of WNS or deaths and showed stable bat populations. The 3 scenarios indicate that studying *G*. *destructans* in bats in Europe and the United States is necessary to understand and control this disease.

Another fungus, *Batrachochytrium dendrobatidis*, is the etiologic agent responsible for chytridiomycosis (*11*), which currently threatens >50% of all amphibian species and is primarily responsible for the global decrease and extinction of >200 amphibian species in the past decade (*12*). Because bats account for >20% of mammalian diversity (*2*) and play major roles in ecosystem functions, we need to understand, monitor, and control the progression of WNS. Otherwise, we may be faced with similar unprecedented and irreversible losses of mammalian biodiversity and entire ecosystems. Because bats control insect populations throughout the world (*13**–**15*), a large decrease in bat populations would result in insect proliferations that would damage agricultural crops and spread many insect-borne diseases.

## Supplementary Material

Technical Appendix White-Nose Syndrome Fungus (Geomyces destructans) in Bat, France
