# Supplementary material for: White-Nose Syndrome Fungus (Geomyces destructans) in Bat, France
Source: Emerg Infect Dis. 2010 Feb;16(2):290–3. doi: 10.3201/eid1602.091391 (PMC2958029; doi:10.3201/eid1602.091391)
Supplement: Technical Appendix — White-Nose Syndrome Fungus (Geomyces destructans) in Bat, France [file 09-1391_Techapp-s1.pdf]

White-Nose Syndrome Fungus (*Geomyces destructans*) in Bat, France

Technical Appendix

Colonies of the fungus on malt extract agar were initially white but with spore production and age, quickly darkened from the center to a dull gray, often with a faint green hue. Colonies were compact and domed, reaching diameters of ≈16 mm in 24 days at 10°C. Hyphae were 2–3 µm in diameter and septate. Older colonies had a strong moldy smell, and brown droplets were exuded on their surfaces. The under surface of colonies also darkened centrally with age, from brown to dark brown. Spore production was prolific from an early stage of growth, giving the colonies a powdery texture; clumps of white mycelia were also occasionally present on colonies.

Spores were typically produced from the tip of groups of short branches, sometimes in loose verticils, at the apices of conidiophores. Spores also developed from side branches and directly from the conidiophore surface. The spores were produced either in small clusters, in short chains of 2–3 spores, or singly. They were hyaline, irregularly curved, broadly crescent-shaped (typically 6–8 µm long and 3–4 µm wide) narrowed at each end, one of which was broadly truncate, often with an annular frill. In side view, many spores appeared obovoid; intercalary spores were barrel-shaped. Spore walls were relatively thick and an outer surface granulation was evident on some.

Microscopic examination of the original swab sample had shown numerous spores having the above-mentioned features. The psychrophilic nature of the fungus and its morphologic features, lead us to conclude that this fungus is *Geomyces destructans*, which was recently isolated from bats in the northeastern United States that had white-nose syndrome.

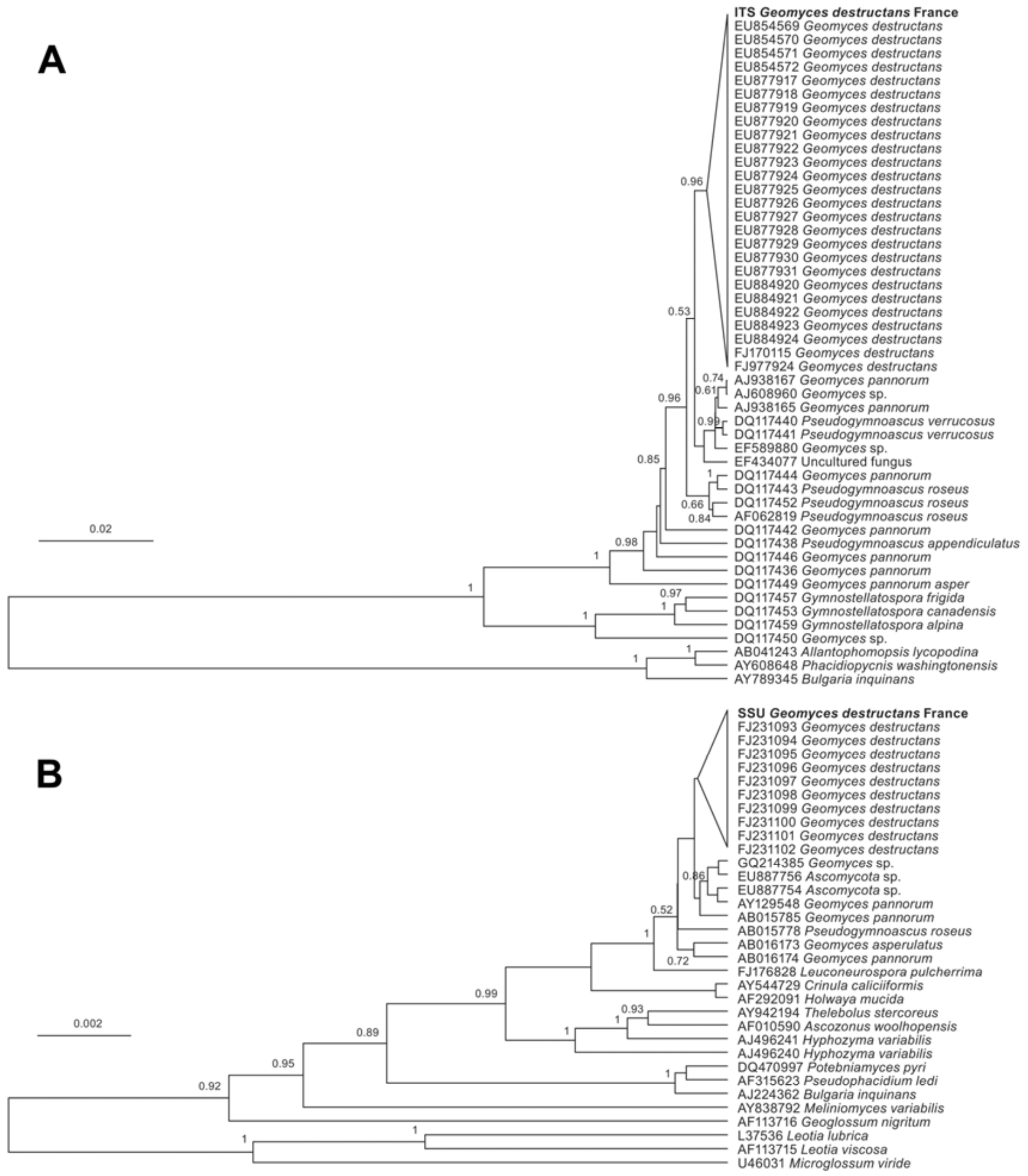

Figure. Phylogenetic relationship between *Geomyces destructans* and other fungus species. A) Bayesian tree for internal transcribed spacer (ITS) alignment (474 nt, Hasegawa, Kishino, and Yano + Γ model). B) Bayesian tree for small subunit (SSU) rRNA alignment (1,865 nt, general time reversible + Γ + invariant sites model). Dendrograms were constructed by using Bayesian analyses with BEAST software (http://beast.bio.ed.ac.uk/Main_Page). Most appropriate substitution models were selected according to ModelTest version 3.7 (http://darwin.uvigo.es/software/modeltest.html). A strict molecular clock model was applied. No outgroup was specified and the constant population size coalescent was used as a tree prior. The program was run for 10,000,000 generations and sampled every 500. The first 1,000,000 generations were discarded as burn-in. Effective sample sizes for the estimated parameters and posterior

probability as calculated with the program Tracer version 1.4 (http://tree.bio.ed.ac.uk/software/tracer) were >800. Bayesian posterior probability values are shown near each supported node (>0.5). The fungus strain identified in this study is shown in **boldface**. Scale bars indicate nucleotide substitutions per site.
